# Indoxyl Sulfate—Review of Toxicity and Therapeutic Strategies

**DOI:** 10.3390/toxins8120358

**Published:** 2016-11-30

**Authors:** Sheldon C. Leong, Tammy L. Sirich

**Affiliations:** The Departments of Medicine, VA Palo Alto HCS and Stanford University, Nephrology 111R, Palo Alto VAHCS, 3801 Miranda Ave., Palo Alto, CA 94304, USA; sleong1@stanford.edu

**Keywords:** indoxyl sulfate, dialysis, uremia

## Abstract

Indoxyl sulfate is an extensively studied uremic solute. It is a small molecule that is more than 90% bound to plasma proteins. Indoxyl sulfate is derived from the breakdown of tryptophan by colon microbes. The kidneys achieve high clearances of indoxyl sulfate by tubular secretion, a function not replicated by hemodialysis. Clearance by hemodialysis is limited by protein binding since only the free, unbound solute can diffuse across the membrane. Since the dialytic clearance is much lower than the kidney clearance, indoxyl sulfate accumulates to relatively high plasma levels in hemodialysis patients. Indoxyl sulfate has been most frequently implicated as a contributor to renal disease progression and vascular disease. Studies have suggested that indoxyl sulfate also has adverse effects on bones and the central nervous system. The majority of studies have assessed toxicity in cultured cells and animal models. The toxicity in humans has not yet been proven, as most data have been from association studies. Such toxicity data, albeit inconclusive, have prompted efforts to lower the plasma levels of indoxyl sulfate through dialytic and non-dialytic means. The largest randomized trial showed no benefit in renal disease progression with AST-120. No trials have yet tested cardiovascular or mortality benefit. Without such trials, the toxicity of indoxyl sulfate cannot be firmly established.

## 1. Background

Indoxyl sulfate is one of the most extensively studied solutes that accumulates in the plasma when the kidneys fail. Originally called “indican,” it was first isolated by Obermayer and Popper in 1911 and noted to be present in high concentrations in the blood of patients with kidney disease [1]. Clinical interest was initially focused on its role in non-renal diseases as a “putrefaction” product of colon microbial metabolism. Studies in the 1950s tested whether the urinary excretion of indoxyl sulfate was associated with a variety of conditions, particularly gastrointestinal and mental disease [2]. Because indoxyl sulfate was known to be cleared primarily by the kidneys and an assay was available, interest later shifted towards its potential role in kidney disease [3]. Numerous studies have since assessed the contribution of indoxyl sulfate to the adverse effects of kidney disease. This review will summarize the evidence for its toxicity. It will also describe the maneuvers which have been attempted to reduce indoxyl sulfate plasma levels and thereby alleviate potential toxic effects.

## 2. Characteristics

Indoxyl sulfate is a small solute with a molecular weight of 213 g/mol and is at least 90% bound to plasma proteins. The description of its protein binding was first reported in studies investigating the reduced drug binding caused by endogenous solutes in uremic plasma [4,5,6]. Being bound to proteins affects the dialytic behavior of indoxyl sulfate. Vanholder et al. [7] were among the first investigators to emphasize that protein-bound solutes including indoxyl sulfate behaved differently than urea during dialysis. They found that the plasma levels of indoxyl sulfate declined less than urea after dialysis and therefore proposed that the protein binding of indoxyl sulfate limited its clearance.

### 2.1. Dialytic and Renal Clearance

Because of its tight protein binding, the hemodialytic clearance of indoxyl sulfate is very low compared to urea, as only the free unbound solute can diffuse across the dialyzer membrane [8,9,10]. During conventional treatment, the clearance of indoxyl sulfate ranges 25–30 mL/min whereas the clearance of urea is higher than 200 mL/min [9,11,12].

The native kidneys, in contrast, achieve very high clearances of indoxyl sulfate through tubular secretion, a function that is not replicated by dialysis [13]. For solutes bound to plasma proteins, the kidneys can achieve clearances that exceed the renal plasma flow by tubular secretion. Protein-bound solutes exist in rapid equilibrium between the bound and free, unbound state. As indoxyl sulfate passes through capillaries surrounding the proximal tubules, the unbound solute is taken up in tubule cells by organic anion transporters (OAT1 and OAT3) located on the basolateral membrane [14,15,16]. It then passes into the tubular lumen through apical membrane transporters which may include the multi-drug resistance protein 4 and breast cancer resistance protein [17,18]. As an unbound indoxyl sulfate molecule is secreted, another molecule will dissociate from plasma protein to maintain the binding equilibrium, allowing for its secretion.

The dialytic clearance of indoxyl sulfate is much lower than the native kidney clearance because dialysis does not replicate tubular secretion. The dialytic clearance of urea, in contrast, is higher than the native kidney clearance because urea is reabsorbed by the kidneys. Therefore, the plasma level of indoxyl sulfate rises to a higher degree than urea in hemodialysis patients relative to normal [19].

### 2.2. Production

Early investigators proposed that indoxyl sulfate was a product “intestinal putrefaction of dietary proteins” [20,21]. Dietary tryptophan that reaches the colon is converted to indole by resident microbes and absorbed into the systemic circulation. Indole is further metabolized by the liver to form indoxyl sulfate, which is then cleared by the kidneys through tubular secretion as described above. The role of colon microbes in producing indoxyl sulfate was described by Brummer and Kasanen in 1955 [22]. They demonstrated that administration of broad spectrum antibiotics led to lower urinary excretion of indoxyl sulfate, presumably due to the reduction of indole-forming colon microbes. More recent studies using untargeted mass spectrometry have shown lower levels of indoxyl sulfate in the plasma of germ free rats compared to conventional rats and in the plasma of hemodialysis patients who have had colectomies compared to those with intact colons [23,24]. The role of the liver in producing indoxyl sulfate was shown by Houssay in 1936 [25]. He infused indole in to dogs that had their digestive tracts removed and dogs that had their livers removed. He found that the dogs with surgically-removed digestive tracts given indole still had elevated indoxyl sulfate plasma levels, whereas dogs with hepatectomies did not. A more recent study by Lin et al. [26] confirms the role of the liver in indoxyl sulfate production. They found that cirrhosis limited the increase in plasma indoxyl sulfate levels in patients with CKD.

Diet also plays an important role in the production of indoxyl sulfate. As indoxyl sulfate is derived from breakdown of tryptophan, higher dietary protein intake increases its production. Subjects with normal kidney function who consumed a high protein diet for 2 weeks had greater indoxyl sulfate level and urinary excretion than those who consumed a low protein diet [27]. In addition, subjects who consumed vegetarian diets had lower indoxyl sulfate excretion than those consuming an unrestricted diet with higher protein content [28].

## 3. Evidence for Toxicity

Multiple studies have suggested that indoxyl sulfate is toxic, having both renal and non-renal effects [21,29]. It has been most extensively identified as a contributor to renal disease progression and vascular disease [21]. The majority of studies, however, have assessed indoxyl sulfate’s toxicity in cultured cells and animal models and its toxicity in humans has not yet been conclusively established. Studies in humans have associated high indoxyl sulfate levels with various adverse outcomes, as summarized in Table 1. This section will describe the reported effects of indoxyl sulfate on renal disease progression, vascular disease, bone disease, and uremic symptoms.

### 3.1. Renal Disease Progression

#### 3.1.1. Pre-Clinical Studies

Indoxyl sulfate has been reported to injure the proximal tubule cells. As described above, indoxyl sulfate is cleared by tubular secretion. As indoxyl sulfate accumulates in the plasma with renal insufficiency, the level in the proximal tubule cells presumably rises and causes injury. Indeed, Enomoto et al. [44] demonstrated the presence of indoxyl sulfate in the proximal tubule cells of rats with 5/6th nephrectomies when plasma levels were raised to about 4.8 mg/dL, near the level seen in hemodialysis patients. A series of studies by Niwa et al. tested the effect of oral indoxyl sulfate administration on renal injury in rats with 5/6th nephrectomies [45,46,47]. Compared to control, rats fed oral indoxyl sulfate or its precursor indole for six weeks had lower inulin clearance and increased glomerular sclerosis.

In proximal tubule cell culture studies, indoxyl sulfate induces inflammation and fibrosis [45,48,49,50,51]. However, results of cell studies must be interpreted with caution, as thoroughly described by Vanholder et al. [29]. In humans and animals, cells are exposed to the free level of indoxyl sulfate, which is sometimes not taken into account in cell culture studies when little or no albumin is added to the medium. So although cells have been exposed to total indoxyl sulfate levels comparable to those seen in dialysis patients, in many cases the cells have been exposed to higher concentrations of free indoxyl sulfate. In addition, cultured cells may have limited OAT expression so that intracellular levels of indoxyl sulfate could be lower than seen in patients with renal insufficiency.

#### 3.1.2. Clinical Studies

The renal injuries observed in pre-clinical studies suggested that high indoxyl sulfate levels may cause progression of renal disease. Wu et al. [30] showed an increased risk of progression, defined as dialysis initiation or 50% reduction in eGFR, with higher total indoxyl sulfate levels in patients with CKD Stage 1 to 4. This association, however, was modest and disappeared when corrected for levels of p-cresol sulfate, another uremic solute. Similarly, Lin et al. [31] showed risk of progression to dialysis in patients with CKD Stage 3 to 5, but unlike the previous study, they did not measure p-cresol sulfate levels. Overall, the limited clinical data is not clear evidence that indoxyl sulfate accelerates progression of renal disease. Alternatively, indoxyl sulfate may simply be a surrogate marker for the severity of tubular injury. Attempts to slow CKD progression by reducing indoxyl sulfate levels have been performed with the oral adsorbent AST-120, as further discussed below.

### 3.2. Vascular Injury

#### 3.2.1. Pre-Clinical Studies

Indoxyl sulfate is thought to injure the vasculature through various mechanisms. Dou et al. [52] showed that indoxyl sulfate impairs the proliferation and repair of human umbilical vein endothelial cells. In this study, the investigators appropriately took into account the protein binding of indoxyl sulfate in their model by using albumin-containing media and demonstrated impairment of endothelial cell repair as a potential mechanism of vascular disease. Other investigators showed that indoxyl sulfate may promote worsening of atherosclerotic lesions and thrombosis by inducing vascular smooth muscle cell proliferation [53]. The mechanism by which indoxyl sulfate elicits this effect has been described by Gondouin et al. and Chitalia et al. [54,55,56]. They found that indoxyl sulfate is an agonist for the aryl hydrocarbon receptor on vascular smooth muscle cells. The activation of this receptor inhibits the degradation of tissue factor, an initiator of coagulation, increasing its levels. Gondouin et al. [54] showed that indoxyl sulfate increased tissue factor expression as well as aryl hydrocarbon receptor-regulated genes in endothelial cells. Chitalia et al. [56] further demonstrated that aryl hydrocarbon receptor antagonists reduced tissue factor expression. Results in CKD and hemodialysis patients further showed a correlation between indoxyl sulfate levels and activation of the aryl hydrocarbon receptor and tissue factor expression.

#### 3.2.2. Clinical Studies

A number of studies have investigated the association of indoxyl sulfate with vascular disease. Sato et al. [32] measured levels in patients with known CAD and eGFR averaging 60 mL/min/1.73 m^2^. They found that a greater proportion of the patients with higher total indoxyl sulfate levels had left ventricular dysfunction on echocardiogram compared to those with lower levels. Other studies found that in patients with cardiomyopathy and CKD Stage 1 to 3, higher total indoxyl sulfate levels were associated with risk of hospitalization for heart failure and cardiac death [33]. Indoxyl sulfate has also been associated with higher degrees of coronary artery calcification and cardiac drug-eluting stent re-stenosis [34,35].

In patients with more severe renal impairment, results have been conflicting. Barreto et al. [36] observed greater risk of cardiovascular mortality in CKD patients with increased total indoxyl sulfate levels. The range of renal impairment was wide, from eGFR 90 mL/min/1.73 m^2^ to dialysis-dependent. However, the association was not adjusted for renal function, making it difficult to distinguish whether indoxyl sulfate levels were specifically related to increased mortality or merely a marker for worsened kidney function. Lin et al. [31] demonstrated an increased risk of cardiovascular events but not mortality with higher total indoxyl sulfate levels in patients with CKD Stage 3 to 5. In hemodialysis patients, several studies failed to demonstrate association of indoxyl sulfate levels with cardiovascular mortality [37,38,39,40], but one study showed increased heart failure events [41].

### 3.3. Bone Disease

#### Pre-Clinical Studies

Only a few studies have investigated the contribution of indoxyl sulfate to bone disease. Nii Kono et al. [57] reported that indoxyl sulfate reduced the expression of parathyroid hormone (PTH) receptor in mouse osteoblasts. However, the suppression of PTH was observed at higher indoxyl sulfate levels than seen in dialysis patients. Hirata et al. [58] studied the condition of low bone turnover in renal failure with reduced production of new bone in setting of low PTH levels. They induced low bone turnover by performing parathyroidectomy in rats. The rats fed indole had further reduced bone turnover activity on tibial bone biopsies compared to control rats. Clinical studies have not yet assessed the association of indoxyl sulfate with bone disease.

### 3.4. Uremic Symptoms

#### 3.4.1. Pre-Clinical Studies

Patients with renal failure exhibit neurological symptoms. Cognitive impairment is prominent and thought to be due to the accumulation of solutes in the plasma and the brain. Perhaps the strongest evidence for this assumption is the awakening of patients from uremic coma following dialysis and the marked improvement in symptoms following kidney transplantation [59,60].

Accumulation of solutes in the brain may be due to impaired removal by transporters across the blood-brain-barrier. Some of the transporters responsible for the removal of endogenous solutes from the brain are the same as those in the proximal tubule of the kidney. In particular, investigators have shown that indoxyl sulfate is removed from the brain to the blood through OAT3 [61]. Indeed, levels of indoxyl sulfate were higher in the brain and plasma of patients with renal insufficiency compared to control subjects [62]. Increased levels of indoxyl sulfate in the plasma could also impair the ability of the brain transporters to remove other solutes, as inhibition of related transporters by indoxyl sulfate in kidney and liver cells has been demonstrated [17,63,64,65].

#### 3.4.2. Clinical Studies

Limited studies have related uremic solute levels to cognitive impairment and their results have been inconclusive. Yeh et al. [42] found higher indoxyl sulfate levels were associated with impaired executive function in patients with CKD stage 3. There was no association, however, in the patients with more advanced CKD. A recent metabolomic analysis in hemodialysis patients did not show a relationship between indoxyl sulfate and cognitive impairment [43]. A weakness of relating solutes to cognition is the lack of tests to assess neurologic impairment specific to uremia.

## 4. Maneuvers to Target Indoxyl Sulfate

The evidence for toxicity described above, albeit inconclusive, has prompted efforts to lower indoxyl sulfate plasma levels. Both dialytic and non-dialytic strategies have been employed, as summarized in Table 2.

### 4.1. Increase Removal

Clearance of indoxyl sulfate by dialysis is limited by protein binding, as only the free, unbound solute can diffuse across the membrane. The concentration gradient driving diffusion is therefore governed by the low free solute plasma level. Dialytic clearance can be increased by increasing convection and increasing diffusion.

#### 4.1.1. Increasing Convection

Increasing convection with hemodiafiltration (HDF) has been tested to increase the clearance of indoxyl sulfate [66,67]. The increase in clearance by convection is approximately equal to the added convective flow multiplied by the free fraction of the solute. Addition of 19–21 L convection per session provided clearance values that were not much higher than conventional hemodialysis clearance values reported in other studies [9,12]. The effect of HDF on plasma levels is uncertain and the effect on clinical outcomes has not yet been studied.

#### 4.1.2. Increasing Diffusion

Another strategy to enhance the clearance of indoxyl sulfate is to increase diffusion. This can be achieved by keeping the level on the dialysate side low. Adding a sorbent to the dialysate to maintain low levels increased the clearance of indoxyl sulfate by greater than two-fold in vitro [68]. Another means to keep the level on the dialysate side low is to increase the dialysate flow and dialyzer membrane size [12,78]. Increasing the dialysate flow maintains a low concentration on the dialysate side, analogous to adding a sorbent. A simultaneous increase in dialyzer membrane size allows for maximal solute transport. Applying a sustained increase in dialytic clearance with higher dialysate flow and larger dialyzer membrane for 2 weeks reduced the total and free plasma levels of indoxyl sulfate by about 6% and 16%, respectively [69]. The effect on clinical outcomes has not yet been studied.

Studies testing membranes that combine diffusion and adsorption of solutes, called mixed-matrix membranes, have been performed. Early investigators attempted to remove bound solutes by perfusing blood over activated charcoal [79,80]. The direct contact of blood with charcoal, however, induced coagulation. Mixed-matrix membranes limit this complication by use of a particle-free membrane layer that is in contact with the blood [81]. Studies in vitro have demonstrated that mixed membranes removed a greater proportion of indoxyl sulfate than non-adsorptive membranes [70].

### 4.2. Reduce Production

An alternative strategy to decrease indoxyl sulfate plasma levels is to reduce the production. As described above, indoxyl sulfate is derived from the breakdown of dietary tryptophan by colon microbes. Production can therefore be suppressed by restricting dietary protein intake, manipulating the colon microbial metabolism, or reducing intestinal absorption.

#### 4.2.1. Protein Restriction

Because indoxyl sulfate is derived from tryptophan, its production increases with higher dietary protein intake [27,28]. One means to reduce production is therefore to restrict protein intake. Indeed, before the availability of dialysis, dietary protein restriction was employed to prevent uremic symptoms based on observations that patients were sicker on high protein diets [82,83]. Marzocco et al. [71] measured total indoxyl sulfate plasma levels in CKD patients with average CrCl 30 mL/min who participated in a cross-over study testing the effect of protein intake on FGF23 levels. They found that total indoxyl sulfate levels were about 37% lower after 1 week of 0.3 g/kg/day protein intake supplemented with ketoanalogues compared to 1 week of 0.6 g/kg/day protein intake.

#### 4.2.2. Manipulate Colon Microbial Metabolism

A major concern with protein restriction is malnutrition [82,84]. An alternative means to reduce indoxyl sulfate production is to manipulate the colon microbial metabolism. This can be potentially achieved by increasing dietary fiber intake. Fiber is a term that describes carbohydrates which are resistant to digestion in the small intestine and are therefore delivered to the colon intact. Fiber is broken down by the colon microbes into short chain fatty acids, which supply energy to the host and microbes [85]. With the increased energy, amino acids that are delivered to the colon may be incorporated into bacterial proteins instead of being broken down into uremic solutes such as indoxyl sulfate.

Increasing fiber intake has resulted in modest if any reduction in indoxyl sulfate levels. Poesen et al. [72] recently tested the effect of increased dietary fiber in the form of arabinoxylan oligosaccharide for 4 weeks versus control starch in CKD patients with average eGFR 33 mL/min/1.73 m^2^. They found no effect on both indoxyl sulfate total plasma levels and urinary excretion. A non-randomized trial in hemodialysis patients similarly showed no change in indoxyl sulfate total plasma levels after 4 weeks of oligofructose-inulin [73]. In contrast, a randomized trial in hemodialysis patients demonstrated a reduction in both the total and free indoxyl sulfate plasma levels by about 18% and 27%, respectively, after 6 weeks of high-amylose corn starch [74]. The discrepancy of these findings may stem from the different forms and doses of fiber as well as the duration of intake.

Some small studies have shown reduction of indoxyl sulfate levels with probiotics, which are available as various strains of bacterial organisms [86,87,88]. Rossi et al. [75] recently tested a combination of fiber and probiotics, termed synbiotics, on indoxyl sulfate levels in CKD patients with average eGFR 24 mL/min/1.73 m^2^ compared to placebo. They found no change in indoxyl sulfate levels after 6 weeks of treatment.

#### 4.2.3. Reduce Intestinal Absorption—AST-120

The most extensive data by far have been from studies of AST-120, a carbon adsorbent administered orally. AST-120 is presumed to bind indole in the colon, thereby preventing its absorption and eventual conversion to indoxyl sulfate. Initial studies showed that AST-120 reduced indoxyl sulfate plasma levels in animals and humans with renal insufficiency [89,90,91,92].

These results motivated a large randomized trial of AST-120, the Evaluating Prevention of Progression in CKD (EPPIC) [76]. Approximately 2000 patients with CKD stage 3 to 5 were randomized to 9 g/day of AST-120 versus placebo. AST-120 did not prevent CKD progression, defined as dialysis initiation, transplantation, and doubling of serum creatinine level. Indoxyl sulfate levels, however, were not measured during the study. A smaller randomized study in patients with CKD stage 3 to 4 similarly showed no benefit in CKD progression with AST-120 [77]. Indoxyl sulfate levels were measured in this study, and there was no significant difference in the change in levels between the AST-120 and placebo groups. Therefore, the lack of benefit in these studies may have been due to the failure to reduce the plasma levels of indoxyl sulfate.

## 5. Conclusions

Pre-clinical studies have demonstrated multiple adverse effects of indoxyl sulfate in various cell lines and animal models. Indoxyl sulfate is present at low levels in subjects with normal kidney function and could have an as yet unidentified physiologic role. There is substantial evidence, however, that it is toxic when it accumulates in conditions of renal insufficiency. Clinical studies have associated increased indoxyl sulfate levels with vascular disease, renal disease progression, and cognitive impairment. The suggested toxicity has prompted efforts to lower the burden of indoxyl sulfate exposure through dialytic and non-dialytic strategies. The largest randomized trial showed no benefit in CKD progression with AST-120 therapy. No trials have yet tested cardiovascular or mortality benefits. Without such trials, the contribution of indoxyl sulfate to the ill effects of renal disease cannot be firmly established.

## Figures and Tables

**Table 1:** Summary of clinical association studies.

Outcome Studied	Study	Types of Patients	Number of Patients	Indoxyl Sulfate Form	Results
Progression	Wu et al. [30]	CKD Stage 1 to 4	268	Total Level	Association with progression (defined by 50% eGFR reduction or dialysis) *
	Lin et al. [31]	CKD Stage 3 to 5	70	Total Level	Association with progression (defined as dialysis)
CV	Sato et al. [32]	Avg. eGFR 60 mL/min/1.73 m^2^	204	Total Level	Association with left ventricular dysfunction
	Shimazu et al. [33]	CKD Stage 1 to 3	76	Total Level	Association with hospitalization for heart failure and cardiac death
	Hsu et al. [34]	Avg. eGFR 66 mL/min/1.73 m^2^	191	Total Level	Association with coronary atherosclerosis
	Tsai et al. [35]	Avg. eGFR 79 mL/min/1.73 m^2^	214	Free Level	Association with cardiac stent restenosis
	Barreto et al. [36]	CKD Stage 2 to dialysis	139	Total Level	Association with aortic calcification; Association with mortality **
	Lin et al. [31]	CKD Stage 3 to 5	70	Total Level	Association with CV event; No association with mortality
	Melamed et al. [37]	Incident HD	521	Total Level	Association with all-cause mortality; No association with CV mortality
	Shafi et al. [38]	Incident HD	394	Free Level	No association with CV event; No association with CV mortality
	Lin et al. [39]	Prevalent HD	100	Total and Free Level	No association with CV mortality
	Shafi et al. [40]	Prevalent HD	1276	Total and Free Level	No association with CV mortality
	Cao et al. [41]	Prevalent HD	258	Total Level	Association with heart failure event
CNS	Yeh et al. [42]	CKD Stage 3 to 5	199	Total Level	Association with cognitive impairment in Stage 3 patients; No association with cognitive impairment in Stage 4 or 5 patients
	Tamura et al. [43]	Prevalent HD	321	Total Level	No association with cognitive impairment

CV: cardiovascular, CNS: central nervous system. * The association disappeared with adjustment for p-cresol sulfate levels; ** the association was not adjusted for baseline eGFR.

**Table 2:** Maneuvers to target indoxyl sulfate.

Strategy	Study	Study Design	Intervention/Duration	Types of Patients	Number of Patients	Results
Increase dialytic removal	Meert et al. [66]	Prospective	HDF (~19 L/session)/9 weeks	Prevalent HD	13	No change in total and free plasma levels compared to baseline
	Krieter et al. [67]	Cross-over	HDF (~21 L/session)/1 week	Prevalent HD	8	No change in dialytic clearance
	Meyer et al. [68]	in vitro	Add dialysate sorbent	n/a	n/a	2.4-fold increase in dialytic clearance
	Camacho et al. [69]	Cross-over	Increase dialysate flow and dialyzer membrane size/2 weeks	Prevalent HD	14	6% reduction in total plasma level; 16% reduction in free plasma level
	Tijink et al. [70]	in vitro	Mixed-matrix membrane (diffusion and adsorption)	n/a	n/a	82% reduction in total level
Suppress production	Marzocco et al. [71]	Post-hoc analysis *	Protein intake 0.3 g/kg/day vs. 0.6 g/kg/day/1 week	CKD(avg. CrCl 30 mL/min)	32	37% reduction in total plasma level
	Poesen et al. [72]	Cross-over	Arabinoxylan vs. control/4 weeks	CKD(avg. eGFR 33 mL/min/1.73 m^2^)	40	No change in total plasma level
	Meijers et al. [73]	Prospective	Oligofructose-inulin/4 weeks	Prevalent HD	22	No change in total plasma level
	Sirich et al. [74]	Randomized	High-amylose corn starch vs. control/6 weeks	Prevalent HD	40	18% reduction in total plasma level; 27% reduction in free plasma level
	Rossi et al. [75]	Randomized	Synbiotic vs. control/6 weeks	CKD (avg. eGFR 24 mL/min/1.73 m^2^)	31	No change in total plasma level
	Schulman et al. [76]	Randomized	AST-120 9 g/day vs. control/avg. follow-up 90 weeks	CKD stage 3 to 5	2028	No benefit in CKD progression
	Cha et al. [77]	Randomized	AST-120 6 g/day vs. control/36 months	CKD stage 3 to 4	538	No benefit in CKD progression; No change in total plasma level between groups

* Measurements of indoxyl sulfate were performed in patients who participated in a cross-over trial testing the effect of protein intake on FGF23 levels. Patients in the protein intake 0.3 g/kg/day group were also supplemented with keto-analogues.
